# Recurrent mediastinal paraganglioma: a Case Report with a long-term follow-up

**DOI:** 10.3389/fmed.2026.1790450

**Published:** 2026-03-25

**Authors:** Wensong Shi, Dan Yang, Huijuan Fang, Zhihua Liang, Guotao Chang, Menghao Zhou, Liangyi Dong, Huiyu Zheng

**Affiliations:** 1 Department of Thoracic Surgery, The Fifth Clinical Medical College of Henan University of Chinese Medicine (Zhengzhou People’s Hospital), Zhengzhou, Henan, China; 2 Department of Oncology, The Fifth Clinical Medical College of Henan University of Chinese Medicine (Zhengzhou People’s Hospital), Zhengzhou, Henan, China; 3 Department of Pathology, The Fifth Clinical Medical College of Henan University of Chinese Medicine (Zhengzhou People’s Hospital), Zhengzhou, Henan, China; 4 Department of Medical Imaging, The Fifth Clinical Medical College of Henan University of Chinese Medicine (Zhengzhou People’s Hospital), Zhengzhou, Henan, China

**Keywords:** long-term follow-up, mediastinal paragangliomas, surgical resection, targeted therapy, tumor metastasis

## Abstract

Mediastinal paraganglioma is a clinically rare neuroendocrine neoplasm. For suspected cases, biochemical detection of plasma and urinary catecholamines and their metabolites is recommended, combined with multimodal imaging (e.g., PET/CT with different tracers) to determine tumor functional status and metastasis. Complete surgical resection remains the treatment of choice; preoperative transcatheter arterial embolization may be considered for highly vascularized tumors to reduce intraoperative bleeding and improve R0 resection rates. Lifelong regular follow-up is advised postoperatively, and sunitinib therapy represents a viable option for recurrent or metastatic disease. This article reports the clinical course of a patient who developed recurrence and metastasis during long-term postoperative follow-up, aiming to enhance clinical understanding of this entity.

## Introduction

Paragangliomas are rare neuroendocrine tumors originating from extra-adrenal chromaffin cells and are also referred to as ectopic pheochromocytomas. Mediastinal paragangliomas account for approximately 2% of all paragangliomas (1) and can be classified into sympathetic and parasympathetic paragangliomas based on their origin, with the mediastinum being the most common site of occurrence (2). Depending on whether they secrete catecholamines and cause clinical symptoms, paragangliomas are further categorized into non-functional and functional subtypes (3, 4). Surgical resection is the conventional management approach upon diagnosis (5, 6). However, due to their complex anatomical location, tumor intricacy, and particularly the intraoperative blood pressure fluctuations caused by catecholamine release in functional paragangliomas, the surgical risk is significantly elevated (7–11). The literature predominantly consists of case reports, and clinical physicians often have limited awareness of these tumors, leading to a high rate of misdiagnosis (12). Moreover, long-term follow-up and recurrence cases are rarely discussed. This article presents a case of mediastinal paraganglioma with postoperative recurrence and pulmonary metastasis, which achieved stable disease after treatment, aiming to enhance clinical physicians’ understanding of mediastinal paragangliomas.

## Case presentation

A 52-year-old female patient presented on April 17, 2020, with an 8-day history of fever. Eight days prior, she developed fever without any obvious precipitating factors, with a peak temperature of 38.9 °C. During the febrile episodes, she did not experience chills, rigors, or fatigue, nor did she have sore throat, abdominal pain, urinary frequency, urgency, or dysuria. She also had no joint pain, recurrent rashes, or oral ulcers. Self-administration of antipyretic medications was ineffective. She was treated with intravenous levofloxacin at an outside hospital, but persistent low-grade fever remained. She was subsequently referred to our hospital for further diagnosis and treatment. The outpatient department admitted her to the Hematology Department with a working diagnosis of “fever of unknown origin.” Since the onset of illness, the patient has remained conscious and in good spirits, with normal diet and sleep, and no significant weight loss or abnormalities in bowel or bladder function.

### Past medical history

The patient has been in good general health. At the age of 43, she underwent surgical excision of a breast nodule, which was pathologically diagnosed as a fibroadenoma of the breast. She denies any history of hypertension, diabetes mellitus, coronary heart disease, or significant trauma. She also denies any history of infectious diseases such as hepatitis or tuberculosis. She has a history of drug allergy, with a positive skin test reaction to cefradine. Her personal history is unremarkable; she denies smoking or alcohol use. She was married at the age of 26 and has one daughter. There is no family history of malignancy.

**Figure 1:**
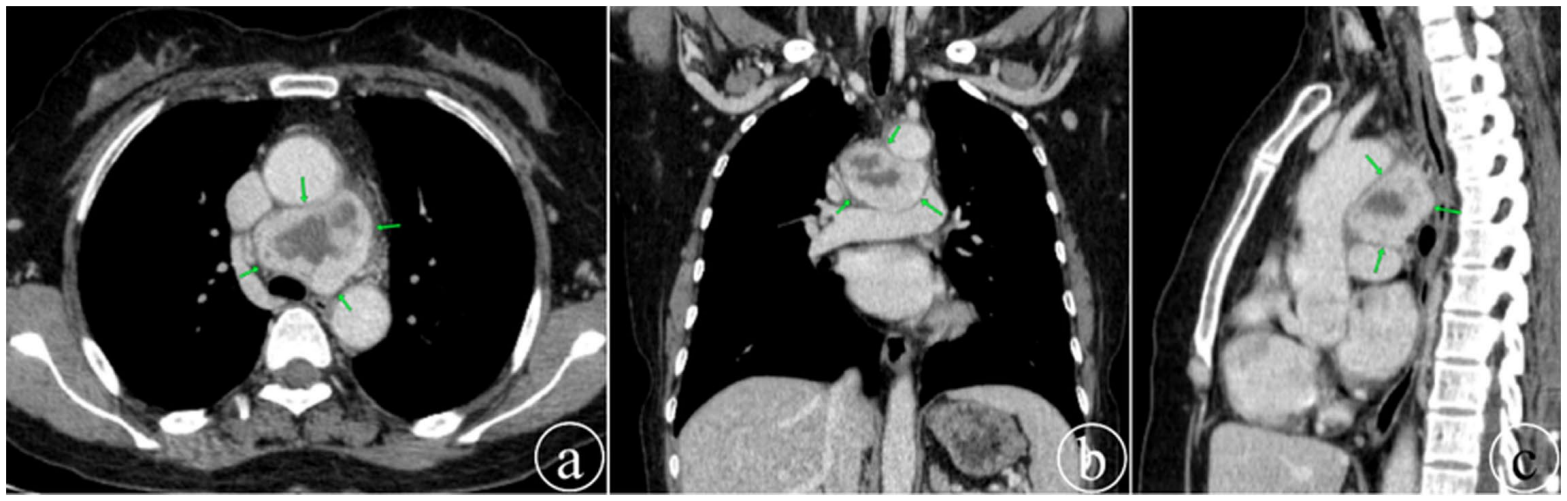
Contrast-enhanced chest CT during the initial visit shows a mediastinal mass in the venous phase (arrow). Panels **(a–c)** display axial, coronal, and sagittal views, respectively, revealing heterogeneous density of the mass and its close relationship with the heart and great vessels.

Upon admission, biochemical tests revealed no significant abnormalities. Routine tumor marker assays, including carcinoembryonic antigen (CEA), cytokeratin fragment 19 (CYFRA 21-1), squamous cell carcinoma antigen (SCC), carbohydrate antigens (CA-125, CA153, CA199, CA724), and neuron-specific enolase (NSE), were all negative. Contrast-enhanced chest CT at admission (Figure 1) showed a mass-like area of abnormal density in the mediastinum, measuring approximately 50 × 36 mm. Multi-phase enhancement revealed marked heterogeneous enhancement within the lesion, with visible patchy low-density areas, suggesting a high likelihood of a neoplastic lesion.

After consultation with the thoracic surgery department, the patient was transferred and underwent right thoracotomy for mediastinal mass resection on April 27, 2020. Preoperative evaluation revealed no definite contraindications to surgery. Intraoperatively, the lesion was found to be located below the aortic arch and above the pulmonary artery, measuring approximately 5 cm in size, with enlarged lymph nodes in the vicinity. The hemiazygos vein was ligated and transected. The mass was dissected using a combination of electrocautery and ultrasonic scalpel for both blunt and sharp dissection, with the ultrasonic scalpel used to transect feeding vessels. The total intraoperative blood loss was approximately 1,000 mL, with transfusion of 4 units of packed red blood cells and 400 mL of plasma. Intravenous fluid administration totaled approximately 1,000 mL. The operation lasted 2 h and 50 min. Postoperatively, the patient was transferred to the intensive care unit and stabilized before being moved to a general ward after 1 day. She had an uneventful recovery and was discharged on postoperative day 8.

**Figure 2:**
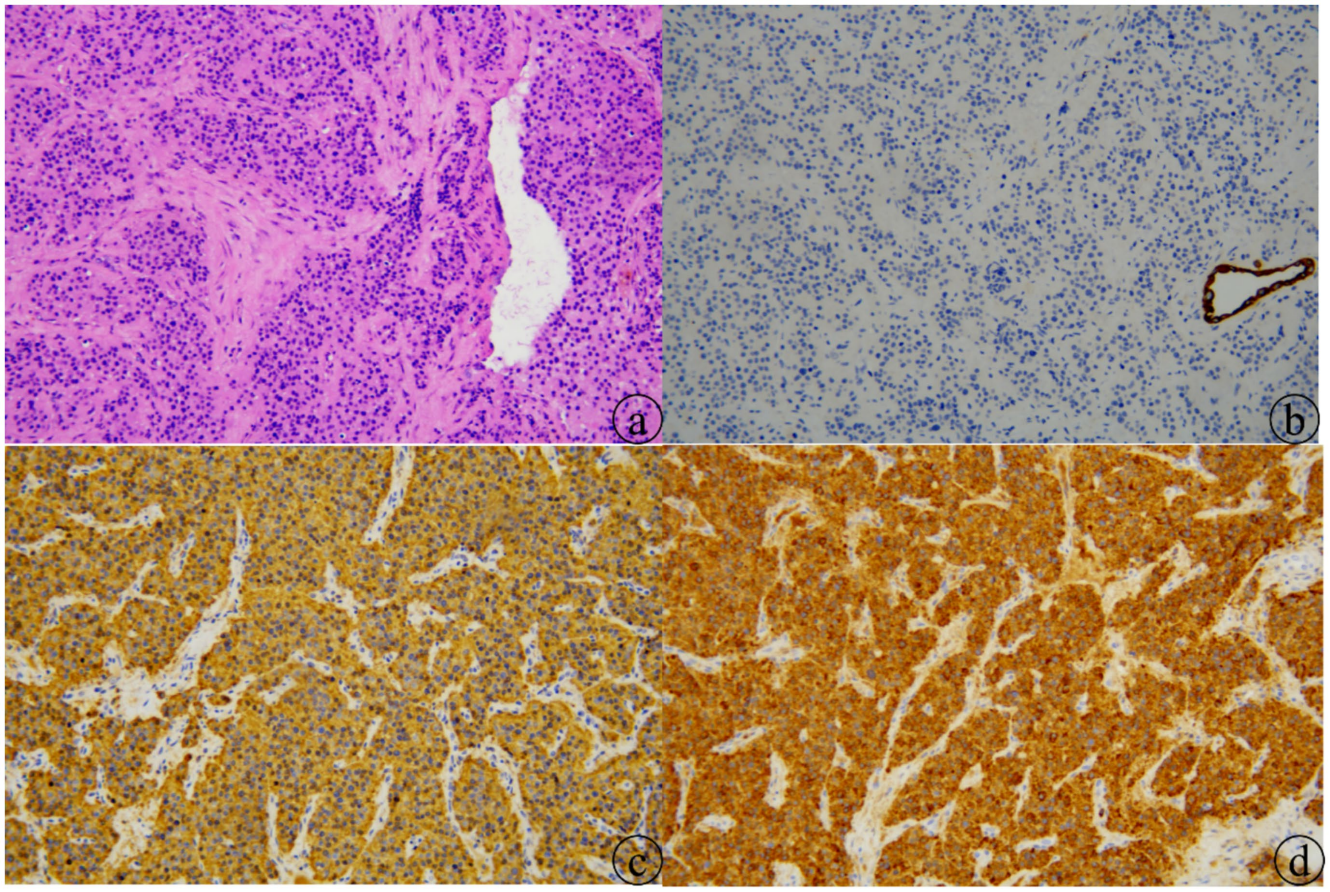
Paraganglioma pathology (×20): **(a)** tumor cells are uniform in size with abundant cytoplasm, arranged in nests separated by patent or obliterated sinusoids (HE). **(b)** Immunohistochemistry: CK negative (SP method). **(c)** Immunohistochemistry: Syn diffusely positive (SP method). **(d)** Immunohistochemistry: CgA diffusely positive (SP method).

Histopathological examination of the resected specimen revealed cellular nests and organoid structures with abundant interstitial sinusoids. The tumor cells had abundant cytoplasm, with some nuclei being enlarged. Extensive hemorrhage and necrosis were observed, and tumor involvement of the capsule was noted. Mitotic figures were rare. Based on the histomorphology and immunohistochemical results, the diagnosis was paraganglioma (Figure 2). Reactive lymphoid hyperplasia was observed in the lymph nodes. Immunohistochemical results were as follows: CK (−), LCA (−), EMA (−), CAM5.2 (−), Vimentin (+), Syn (−), CgA (+), CD56 (+), Ki67 (proliferation index approximately 20%), PLAP (−), CD117 (−), OCT3/4 (−), CD30 (−), TFE3 (−), CD20 (−), CD3 (−), S100 (positive in sustentacular cells).

## Outcome and follow-up

**Figure 3:**
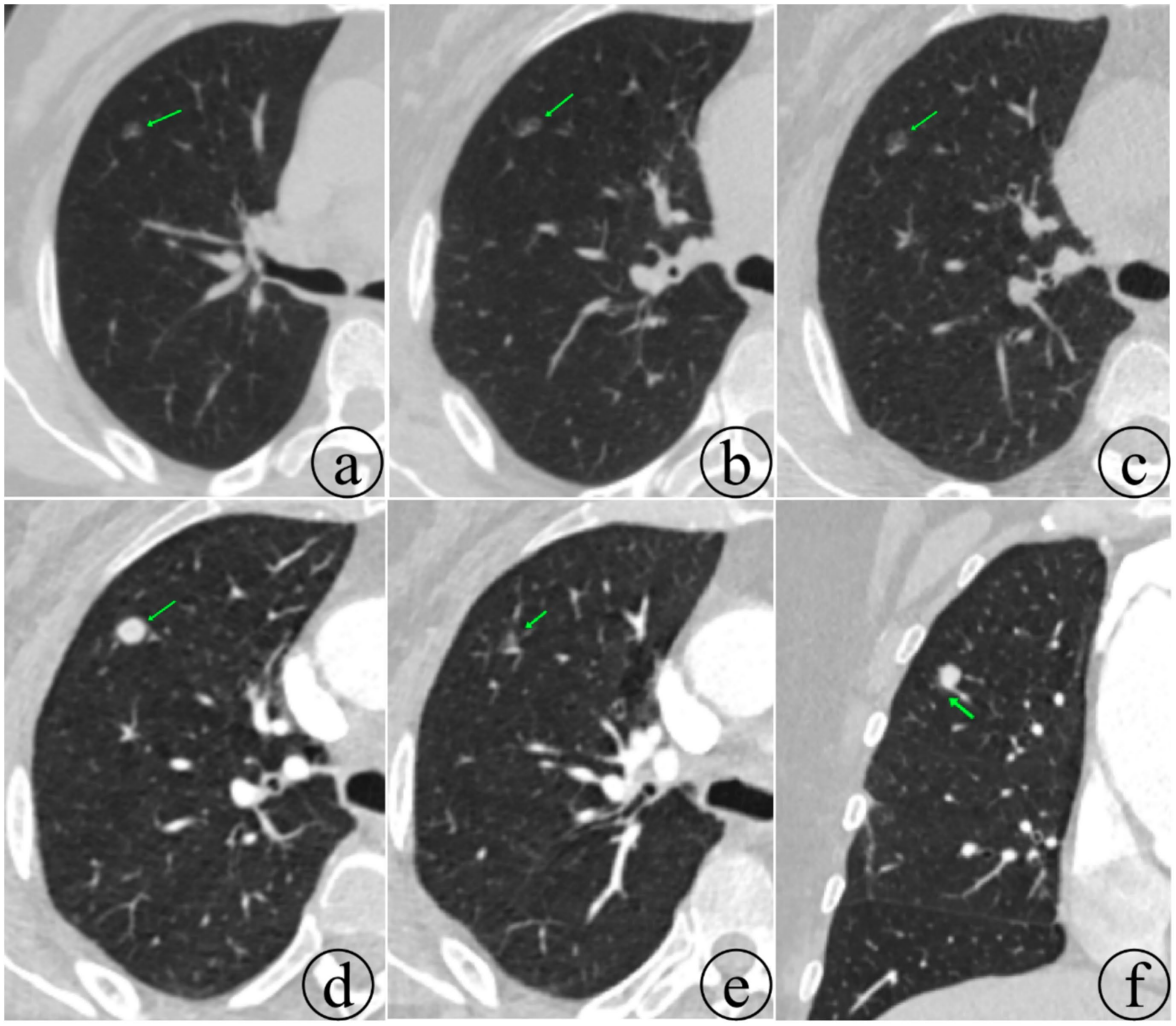
Changes in the nodule of the right upper lobe during follow-up. The nodule is shown to have increased in size and become more solid. However, a ground-glass component is still observed at the inferior margin of the nodule (panels **e,f**) in the examination dated May 13, 2024. The dates of the respective examinations are as follows: **(a)** April 19, 2020; **(b)** August 27, 2020; **(c)** May 30, 2022; and **(d)** May 13, 2024.

The patient did not follow-up regularly after surgery. Imaging findings during follow-up are shown in Figure 3. In May 2024, a chest CT scan revealed a solid nodule in the anterior segment of the right upper lobe, which had become more solid compared with previous records, raising a high suspicion for a neoplastic lesion. Subsequently, the patient underwent a “single-port thoracoscopic wedge resection of the lung + pleural adhesion lysis” at our hospital on May 18, 2024. Intraoperative frozen section pathology showed diffuse or nested cellular growth with mild to moderate atypia and abundant eosinophilic cytoplasm, which did not rule out a neoplastic lesion. The final pathological diagnosis was metastatic paraganglioma, while another nodule was diagnosed as pulmonary carcinoma *in situ* after routine sampling. Immunohistochemical results were as follows: CK (−), LCA (focal +), EMA (−), CAM5.2 (−), Vimentin (+), Syn (+), CgA (+), CD56 (+), S100 (−), Ki67 (approximately 30% +), PLAP (−), CD117 (−), OCT3/4 (−), CD30 (−), TFE3 (−), PD-1 (−), PD-L1 (TPS approximately 1%, clone 22C3).

After a good postoperative recovery, the patient underwent [^18^F]FDG-PET/CT imaging, which revealed changes consistent with postoperative status following resection of mediastinal paraganglioma and right pulmonary nodule. A soft tissue nodule was identified in the mediastinal zone 5 (measuring 1.91 × 1.87 cm) with markedly increased FDG uptake (SUV_max_: 19.61), suggestive of tumor recurrence and in close proximity to the ascending aorta. Multiple round pulmonary nodules were observed bilaterally, with the largest located in the posterior basal segment of the left lower lobe (diameter approximately 0.67 cm) showing increased FDG metabolism (SUV_max_: 2.08), suggestive of metastatic disease. There was an area of increased density in the right upper lobe with elevated FDG uptake. The right pleura was thickened with increased FDG uptake, and subcutaneous soft tissue thickening with increased FDG uptake was noted in the right chest wall. These findings, along with subcutaneous emphysema, pleural effusion, and minimal pneumothorax on the right side, were considered postoperative changes. No other significant abnormalities were detected.

Following a multidisciplinary consultation, the patient was transferred to the oncology department for further treatment. Based on the consultation opinions from the higher-level hospital and the patient’s individual condition, and with the consent of the family, the patient began oral sunitinib therapy on May 31, 2024. The initial dose was 50 mg once daily for 14 consecutive days every 21 days (50 mg qd d1–14 q21d). Due to the development of oral ulcers and edema, the dose was reduced to 37.5 mg once daily. During the treatment, the patient experienced mild leukopenia and thrombocytopenia, which improved after symptomatic treatment. Additionally, the patient developed hypothyroidism and was treated with continuous oral levothyroxine.

**Figure 4:**
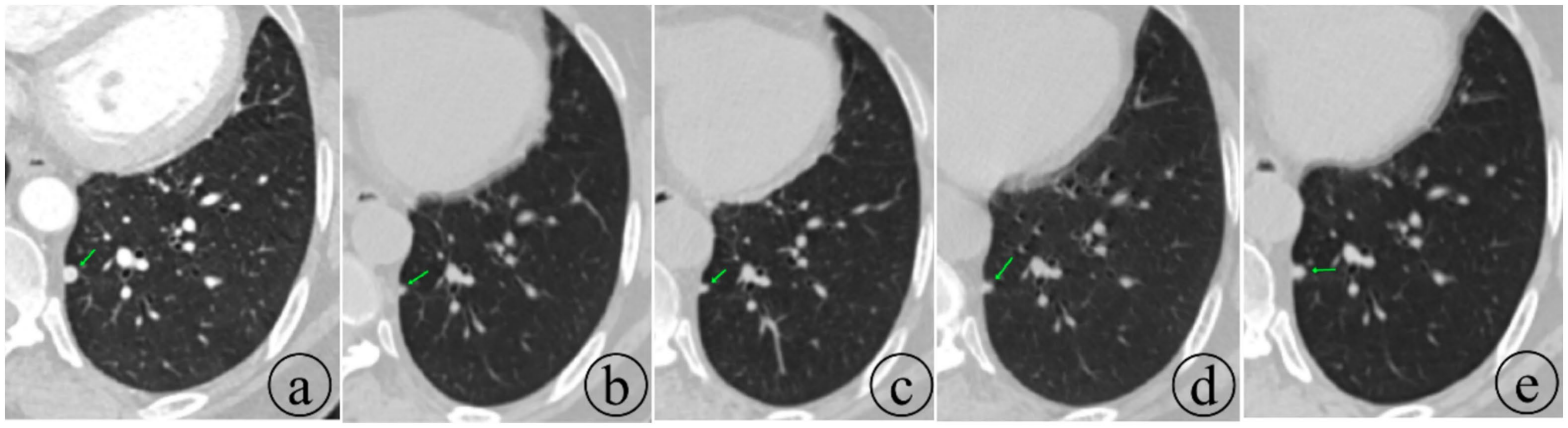
Follow-up changes in the metastatic lesion in the left lower lobe during oral sunitinib therapy. The lesion is shown to have rapidly decreased in size initially but subsequently exhibited a slow increase. The dates of the respective examinations are as follows: **(a)** May 13, 2024; **(b)** July 12, 2024; **(c)** February 17, 2025; **(d)** July 13, 2025; and **(e)** November 4, 2025.

**Figure 5:**
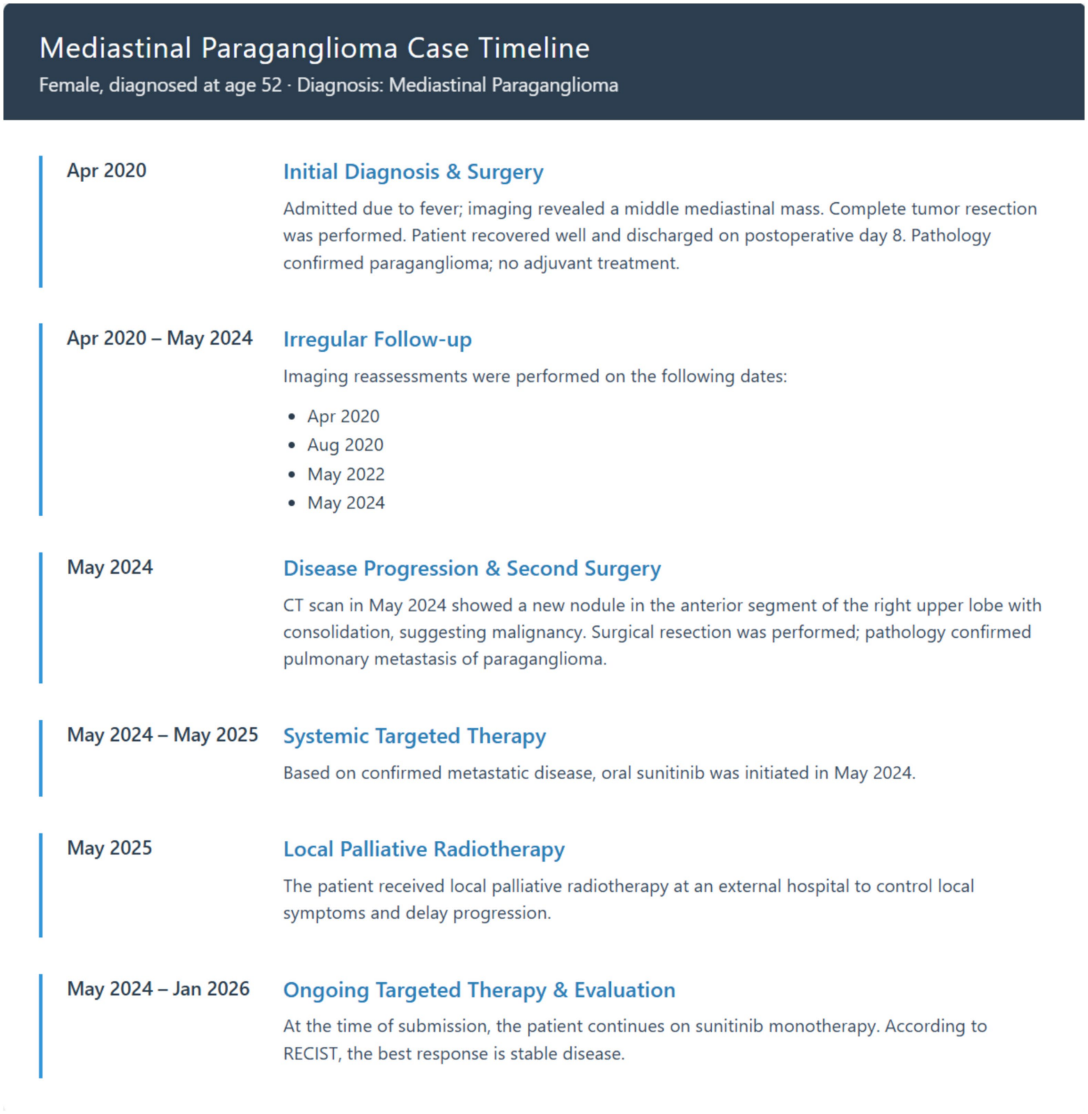
Timeline figure of the clinical information.

On September 13, 2024, the patient underwent [^18^F]DOPA-PET/CT at an outside hospital, which revealed lymph node metastasis below the aortic arch after surgical resection of mediastinal paraganglioma with pulmonary metastasis, with mild increase in DOPA metabolism. The following day (September 14, 2024), the patient had [^68^Ga]SSA-PET/CT imaging. Compared with the DOPA-PET/CT of September 13, 2024, there was a mass shadow below the aortic arch after surgical resection of mediastinal paraganglioma with pulmonary metastasis, suggestive of recurrence. The lesion did not express somatostatin receptors, with uncertain SSTR-RADS uptake and mild increase in DOPA metabolism. Follow-up evaluations in July 2024, September 2024, February 2025, and May 2025 all indicated stable disease. In May 2025, the patient was placed in the supine position and immobilized with a body frame. Target volume delineation and treatment planning were performed for the mediastinal mass. Gamma knife stereotactic radiotherapy was administered with a fractional dose of 300 cGy per fraction, using the 50% isodose line to cover the target. A total dose of 62 Gy was delivered in 16 fractions for local tumor control. Subsequently, the patient continued targeted therapy with sunitinib until the time of manuscript submission. As of the date of submission, the patient’s overall condition remained stable, although a slow increase in size was observed in the metastatic lesion in the left lower lobe of the lung (Figure 4). Although response evaluation criteria in solid tumors (RECIST) assessment demonstrated stable disease, emerging resistance warrants careful monitoring. Figure 5 shows the timeline for the patient.

## Discussion

This case report describes a rare mediastinal paraganglioma that exhibited aggressive biological behavior characterized by local recurrence and distant (pulmonary) metastasis, ultimately achieving stable disease through multidisciplinary comprehensive treatment. Although paragangliomas are generally considered slow-growing tumors, their biological behavior is heterogeneous, with some cases presenting with aggressive features. In this case, the patient developed pulmonary metastasis and local regional recurrence 4 years after complete surgical resection, indicating a certain degree of malignant potential. Certain pathological features identified in the postoperative assessment may provide clues: the presence of extensive hemorrhage and necrosis within the tumor, involvement of the capsule, and a relatively high Ki-67 proliferation index (approximately 20%). Previous studies (13) have shown that a Ki-67 index ≥3% is associated with an increased risk of disease progression, recurrence, or metastasis in patients with paragangliomas. The Ki-67 index of approximately 20% at initial diagnosis in this case is consistent with these findings and may serve as one of the predictive factors for clinical recurrence risk. Additionally, the presence of enlarged lymph nodes surrounding the tumor at the time of initial surgery (although pathologically reactive hyperplasia) may suggest an active immune or inflammatory response within the tumor microenvironment. The disease progression in this case underscores the importance of long-term, close imaging follow-up for paragangliomas with such high-risk pathological features, even after “curative” surgery. In this case, the evolution of pulmonary nodules from absence to presence observed through serial CT scans highlights the significance of regular follow-up.

Clinically, paragangliomas can be categorized into functional and non-functional types based on whether they secrete catecholamines and their metabolites (3, 4). Functional tumors often present with typical symptoms such as hypertension, headache, and palpitations. In this case, the patient initially presented with fever of unknown origin, without the typical symptoms of catecholamine excess. The present patient presented with fever of unknown origin as the initial manifestation, lacking the classic clinical symptoms of catecholamine hypersecretion. However, preoperative plasma or urinary metanephrines were not routinely assessed; consequently, this case should be classified as “clinically silent catecholamine excess.” However, immunohistochemical results revealed that the tumor cells expressed chromogranin A (CgA) and CD56, confirming their neuroendocrine origin. This dissociation between “clinically non-functional” and “pathologically functional” is not uncommon and highlights the importance for clinicians to consider paraganglioma in the differential diagnosis of mediastinal masses, even in the absence of related symptoms, and to perform relevant biochemical screening when possible, in order to avoid the potential risk of catecholamine crisis triggered by tumor manipulation during surgery. Postoperatively, the patient exhibited suboptimal compliance with regular follow-up protocols and received care at multiple medical institutions, resulting in the absence of catecholamine-related biochemical assessments during the surveillance period. Therefore, subsequent follow-up should emphasize the monitoring of catecholamine level fluctuations, and it is recommended that these be evaluated in conjunction with tumor markers to enhance the comprehensiveness of postoperative surveillance.

Imaging plays a central role in the diagnosis, staging, therapeutic evaluation, and follow-up of paragangliomas, with significant clinical value in the diagnosis and initial assessment of paragangliomas, monitoring of recurrence and metastasis, and exploration of therapeutic targets. Contrast-enhanced chest CT clarifies the morphology, location, and vascular relationships of the tumor, providing a basis for surgical planning and holding an important position in diagnosis and initial assessment. The diagnostic process of this case also demonstrated the value of different PET tracers in the diagnosis and treatment of paragangliomas. After the identification of pulmonary nodules, which were confirmed as metastatic by surgery, [^18^F]FDG PET-CT revealed a high-metabolic recurrence in the mediastinal region (with an SUV_max_ as high as 19.61) and multiple metabolically active small nodules in both lungs, providing key information for disease restaging and confirming the systemic nature of the disease. Subsequent [^68^Ga]SSA PET-CT was performed to detect the expression of somatostatin receptors (SSTR) on tumor cells to assess the possibility of peptide receptor radionuclide therapy (PRRT). A study by Fernandez-Pombo et al. (14) showed that [^68^Ga]Ga-DOTA-TOC PET/CT has a higher detection rate and higher average maximum standardized uptake value compared with [^18^F]DOPA PET/CT in detecting multifocal paragangliomas and metastatic lesions. Therefore, when paraganglioma is clinically suspected, especially for differentiating its functionality, [^68^Ga]SSA-PET/CT can be used as a further assessment tool. In this case, the results showed uncertain SSTR uptake, while [^18^F]DOPA PET-CT revealed only mild DOPA metabolic increase in the lesion. This imaging feature of 68Ga-SSA negativity and strong 18F-FDG positivity is often associated with tumor dedifferentiation, higher proliferative activity, and more aggressive clinical behavior, providing an important basis for selecting anti-angiogenic targeted therapy rather than peptide receptor radionuclide therapy (15).

Mediastinal paragangliomas are typically highly vascular and carry a significant risk of intraoperative bleeding. In some cases, surgical resection may be performed with the support of cardiopulmonary bypass (16–18). In this case, the patient experienced substantial intraoperative bleeding, with a total blood loss of approximately 1,000 mL. The patient received transfusions of 4 units of packed red blood cells and 400 mL of plasma. Preoperative interventional tumor embolization may help reduce intraoperative bleeding (19–24), and comprehensive preoperative planning is essential for mitigating such surgical risks.

Regarding the surgical resection of a ground-glass opacity (GGO) nodule that progressed, with the postoperative pathology revealing concurrent pulmonary carcinoma *in situ*, how should this be interpreted? Could the metastatic paraganglioma have originated from the GGO nodule and progressed to a solid nodule? After careful comparison of pathological and imaging findings, it appears that the inferior margin of the solid nodule in the right upper lobe still shows some ground-glass components (see Figures 2e,f). Following multidisciplinary team (MDT) consultation with pathologists and radiologists, we concluded that the solid component and the ground-glass opacity (GGO) represented distinct pathological entities. Their anatomical proximity resulted in mechanical compression of the GGO by the solid component, creating a radiological illusion of a unified lesion rather than indicating that the GGO was initially metastatic and had progressively evolved into a solid nodule. The original GGO nodule is likely to be pulmonary carcinoma *in situ*.

Surgical resection remains the primary treatment modality for paragangliomas. However, for unresectable, recurrent, or metastatic paragangliomas, systemic treatment options are currently limited (25, 26). Sunitinib, a multikinase inhibitor targeting vascular endothelial growth factor receptors, has been used to inhibit tumor angiogenesis. Based on its efficacy in advanced pancreatic neuroendocrine tumors, sunitinib has also been explored for the treatment of progressive paragangliomas (27–30). In this case, the patient achieved stable disease after treatment with sunitinib, demonstrating its efficacy in controlling paraganglioma growth over a certain period. However, adverse effects such as oral ulcers, edema, hematologic toxicity, and hypothyroidism, along with the need for dose adjustment (from 50 mg to 37.5 mg), highlight the challenges of managing tolerability with such agents. More importantly, long-term follow-up revealed a slow increase in size of the metastatic lesion in the left lower lobe, suggesting the potential development of secondary resistance. Resistance to anti-angiogenic therapy in paraganglioma may arise through activation of alternative pro-angiogenic pathways (e.g., FGF, PDGF), HIF-driven pseudohypoxic signaling secondary to SDHx mutations, and adaptive clonal selection under prolonged TKI exposure. These interconnected mechanisms highlight the need for combination strategies targeting multiple resistance pathways simultaneously. This further underscores the need to explore more effective subsequent treatment options for advanced paragangliomas, including other targeted therapies, chemotherapy, or novel combination strategies.

Several limitations were identified in the diagnostic and therapeutic management of this patient. First, preoperative differential diagnosis failed to adequately consider paraganglioma as a potential etiology, and comprehensive evaluation modalities—including PET-CT, plasma and urinary metanephrines assays, and transbronchial needle biopsy—were not performed. Second, preoperative interventional embolization was not undertaken to prevent intraoperative hemorrhage, resulting in substantial bleeding that necessitated emergent blood transfusion. Furthermore, genetic assessment was incomplete, as mutational analysis of the succinate dehydrogenase subunit D (SDHD) and subunit B (SDHB) genes was not conducted to evaluate hereditary predisposition. Finally, postoperative surveillance was suboptimal; regular monitoring of plasma or urinary metanephrines was not implemented to guide clinical decision-making during follow-up, and systematic inquiry into the patient’s family history was neglected. Furthermore, tumor heterogeneity, as a critical determinant of therapeutic response and clinical outcome, should be duly considered in clinical decision-making. The diagnostic and therapeutic experience derived from this case is intended solely as a reference for clinical practice.

## Conclusion

Mediastinal paragangliomas are rare and potentially aggressive tumors. Even after complete surgical resection, patients with high-risk pathological features require lifelong close follow-up. Multimodal imaging, especially PET-CT with different tracers, is invaluable throughout the disease management cycle, aiding in disease staging, monitoring recurrence, and guiding therapeutic choices. For patients with unresectable tumors or postoperative recurrence, targeted therapies such as sunitinib represent important treatment options, but their toxicities and potential for resistance must be closely monitored. This case highlights that a comprehensive, individualized treatment strategy based on multidisciplinary collaboration, encompassing surgery, medical therapy, and radiotherapy, is key to managing such complex cases, extending patient survival, and maintaining quality of life. Future directions to improve long-term patient outcomes include further exploration of the molecular pathogenesis, identification of new prognostic markers, and the discovery of novel therapeutic targets.

## Data Availability

The original contributions presented in the study are included in the article/supplementary material, further inquiries can be directed to the corresponding author.
